# Late hepatitis C virus diagnosis among patients with newly diagnosed hepatocellular carcinoma: a case–control study

**DOI:** 10.1186/s12876-022-02504-6

**Published:** 2022-09-17

**Authors:** Shen-Shong Chang, Hsiao-Yun Hu, Yu-Chin Chen, Yung-Feng Yen, Nicole Huang

**Affiliations:** 1 Division of Gastroenterology, Taipei City Hospital Yang-Ming Branch, Taipei City, Taiwan; 2 Department of Internal Medicine, Taipei City Hospital Yang-Ming Branch, Taipei City, Taiwan; 3 Department of Medicine, School of Medicine, National Yang Ming Chiao Tung University, Taipei City, Taiwan; 4 Department of Public Health, Institute of Public Health, National Yang Ming Chiao Tung University, Taipei City, Taiwan; 5 Institute of Hospital and Health Care Administration, School of Medicine, National Yang Ming Chiao Tung University, Room 201, The Medical Building II, No. 155, Section 2, Li-Nong Street, Taipei 112, Taiwan; 6 Department of Education and Research, Taipei City Hospital, Taipei City, Taiwan; 7 Section of Infectious Diseases, Taipei City Hospital Yang-Ming Branch, Taipei City, Taiwan; 8 Department of Health Care Management, National Taipei University of Nursing and Health Sciences, Taipei City, Taiwan

**Keywords:** Cirrhosis, HCC, Late HCV diagnosis, Diabetes mellitus, Early screening

## Abstract

**Background:**

New direct-acting antiviral therapies have revolutionized hepatitis C virus (HCV) infection therapy. Nonetheless, once liver cirrhosis is established, the risk of hepatocellular carcinoma (HCC) still exists despite virus eradication. Late HCV diagnosis hinders timely access to HCV treatment. Thus, we determined trends and risk factors associated with late HCV among patients with a diagnosis of HCC in Taiwan.

**Methods:**

We conducted a population-based unmatched case–control study. 2008–2018 Claims data were derived from the Taiwan National Health Insurance Research Database. Individuals with an initial occurrence of liver cancer between 2012 and 2018 were included. The late HCV group were referred as individuals who were diagnosed with HCC within 3 years after HCV diagnosis. The control group were referred as individuals who were diagnosed more than 3 years after the index date. We used multivariable logistic models to explore individual- and provider-level risk factors associated with a late HCV diagnosis.

**Results:**

A decreasing trend was observed in the prevalence of late HCV-related HCC diagnosis between 2012 and 2018 in Taiwan. On an individual level, male, elderly patients, patients with diabetes mellitus (DM), and patients with alcohol-related disease had significantly higher risks of late HCV-related HCC diagnosis. On a provider level, patients who were mainly cared for by male physicians, internists and family medicine physicians had a significantly lower risk of late diagnosis.

**Conclusions:**

Elderly and patients who have DM and alcohol related disease should receive early HCV screening. In addition to comorbidities, physician factors also matter. HCV screening strategies shall take these higher risk patients and physician factors into consideration to avoid missing opportunities for early intervention.

**Supplementary Information:**

The online version contains supplementary material available at 10.1186/s12876-022-02504-6.

## Background

Hepatitis C virus (HCV) is a major cause of liver cancer and morbidity and mortality worldwide [1]. In 2005, 123 million people contracted HCV [1]; by 2015, this number increased to more than 185 million [2, 3]. HCV prevalence also increased from approximately 2% in 2005 to up to 4.4% in 2015 in Taiwan [4, 5]. The direct health care costs of HCV-related disease are enormous, and costs are especially high for patients with end stage liver disease [6].

Currently, new HCV treatments are becoming more readily available. New direct-acting antiviral (DAA) therapies are one example of revolutionized therapies for HCV infection. DAA therapies cause fewer adverse effects and allow higher degrees of viral clearance than traditional therapies [7, 8]. Without the development of an effective prophylactic vaccine [9], DAA therapy provides virus prevention by reducing the risk of HCV transmission [10, 11]. Nonetheless, once liver cirrhosis is established, the risk of hepatocellular carcinoma (HCC) still exists despite virus eradication with DAA therapy [12, 13]. Therefore, in addition to researching the epigenetic mechanisms involved in HCV-related HCC [14], it is equally critical to prevent late diagnosis of HCV. Early detection of HCV may improve access to timely HCV treatment and help health care providers overcome the HCC treatment hurdle.

Although HCC surveillance significantly improves early tumor detection, curative therapies, and improvement of survival [15], poor adherence to HCC surveillance guidelines in high-risk patients may lead to lower proportions of HCC diagnosis via screening among retrospective studies, which were closer to real-world clinical practice [16]. Because patients do not often experience early symptoms of HCV, less than 50% of patients notice their HCV infections [17]. Thus, viral infection is initially difficult to diagnose, and it is challenging to estimate its incidence [1]. The progression of the disease varies from person to person and begins with no symptoms during the early stage of infection [18]. HCV infection usually takes 30 or more years to develop into end stage liver disease (ESLD) [18]. Moorman et al. found that 17% of individuals were diagnosed as having HCV and cirrhosis simultaneously; this indicates a late diagnosis of HCV, which obstructs the ability for patients to obtain early interventions and therapies [19]. The difficulty in diagnosing HCV due to lack of symptoms, along with unidentified risk factors of the disease, is likely to lead to late diagnosis of HCV infection and related HCC.

According to previous research, several additional factors may be associated with late HCV-related HCC diagnosis and hinder timely access to appropriate treatments. Illegal drug use [20, 21], older age upon infection [18], less likelihood to exhibit medical care–seeking behavior [22], low income or socioeconomic status (SES) [19], and distance from a health care provider [23] have also been identified as risk factors for late HCV diagnosis. Critically, the literature illustrates that excessive alcohol intake and coinfection with human immunodeficiency virus (HIV) [24] may accelerate the progression of HCV, cause earlier development of cirrhosis or HCC, and increase the risk of late HCC diagnosis, reducing the opportunity for patients to receive timely treatment [18].

Studies have focused on known risk factors such as illegal drug use, alcohol use, SES, physician visits, and residential area [19, 22, 23, 25]. Despite increased knowledge of these risk factors, the rate of late diagnosis is still not optimal. The increasing prevalence of chronic conditions, along with patient and provider behaviors, may be hidden factors contributing to under-diagnosis and late diagnosis of HCV. To identify more hidden and unexplored risk factors for late diagnosis, our research analyzed patient characteristics, such as multiple chronic conditions and routine medical checkups, as well as factors related to the health care environment, such as health care provider characteristics [26, 27]. We examined the trend of late HCV-related HCC diagnosis rates over time and explored individual- and provider-related factors associated with late HCV diagnosis among patients with newly diagnosed HCC under the National Health Insurance program in Taiwan, a typical single-payer universal health insurance plan.

## Methods

### Study design and data source

The main data source of this population-based unmatched case control study was the 2008–2018 National Health Insurance Research Database (NHIRD). The NHIRD contains health care data of 99% of the population of Taiwan and includes comprehensive claims and enrollment information, including demographic data, dates of clinical visits and admission, diagnostic codes, and prescription details. NHIRD used the International Classification of Diseases, 9th Revision, Clinical Modification (ICD-9-CM) to record diagnoses prior to January 2016 and used the 10th Revision (ICD-10-CM) thereafter.

### Study population

**Fig. 1:**
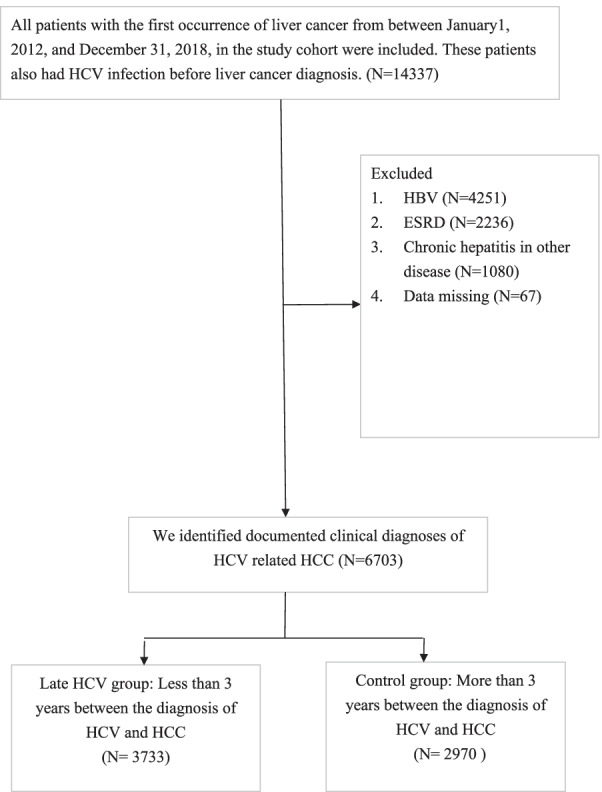
Flow chart for selection of late HCV group and control group. HCV = hepatitis C virus; HCC = hepatocellular carcinoma; HBV = hepatitis B virus; ESRD = end stage of renal disease

We used the NHI claims files to identify individuals with an initial occurrence of liver cancer (ICD-9-CM codes 155.0, 155.2/ICD-10-CM codes C22.0, C22.2, C22.3, C22.4, C22.7, C22.8, C22.9, Z51.12) from January 1, 2012, to December 31, 2018. The eligible participants were divided into two groups based on the amount of time between HCV diagnosis (ICD-9-CM codes V02.62, 070.41, 070.44, 070.51, and 070.54/ ICD-10-CM codes B17.11, B19.21, B18.2, B17.10, B19.20, B18.2, Z22.52) and occurrence of HCC. The late group was defined as having a time lag of 3 years or fewer between HCV diagnosis and HCC diagnosis, including the HCC diagnosis elicited testing and subsequent HCV diagnosis. This indicated delayed diagnosis of HCV infection due to the slow progression nature of HCV [18]. The control group was defined as if individuals were diagnosed with HCV more than 3 years before their HCC diagnosis. Sensitivity analyses were conducted using two alternative time cut-offs (2 years and 5 years) in the definitions of late diagnosis of HCV. We identified 14,337 patients with HCC who had been previously diagnosed with HCV. The exclusion criteria included hepatitis B virus (HBV) infection, end stage renal disease (ESRD), and chronic hepatitis in other disease. After these patients were excluded, we identified 6703 documented clinical diagnoses of HCV-related HCC, 3733 of which were in the late group and 2970 of which were in the control group (Fig. 1).

### Patient characteristics

The demographic data collected were patient age, sex, and comorbidities. Comorbidities were defined as diseases that existed within 3 years before the date of HCV diagnosis. The comorbidities identified in this study were diabetes mellitus (DM), dyslipidemia, psychiatric disorders, alcohol-related disease, and malignancy disease. DM, dyslipidemia, psychiatric disorders including schizophrenia, depression, and anxiety were identified using one inpatient discharge record or at least two outpatient visits with the specific diagnosis and disease-related prescriptions. Alcohol-related diseases were identified using one inpatient discharge record or at least two outpatient visits with the specific diagnosis. Patients with malignancy and hematological malignancy excluding HCC were identified using the NHI catastrophic illness file records. The related ICD-9/10-CM codes were provided in Appendix A (Additional file 1). Health care–seeking behavior included the variables of outpatient utilization and medical checkups. Outpatient utilization was defined as the number of outpatient visits within 3 years preceding the date of HCV diagnosis and was categorized into four levels: very low, low, medium, and high. The routine checkup variable was defined as whether individuals had undergone any routine medical checkup within 3 years preceding the HCV diagnosis. The NHI monthly wage and enrollment category was used as a proxy for SES. People without a well-defined monthly payroll were categorized into two groups: union or association members such as members of farmers’ associations and members of a lower income group such as veterans [28]. We divided SES into four categories: a well-defined monthly payroll of more than NT$60,000, a monthly income of less than NT$60,000, union or association members, and a lower income group (NT: New Taiwan dollars; US$1 = approximately NT$30.24).

### Provider characteristics

Taiwanese people enjoy complete freedom in choosing their care providers under the NHI program [29, 30]. Thus, health care providers were defined as providers who provided most of the medical services to an individual within 3 years before the HCV diagnosis in our analysis. The provider-level characteristics identified in this study were physician age, sex, and specialty. Under the NHI program in Taiwan, hospitals are classified according to their accreditation status, quality, staffing, and infrastructure. Accreditation status was divided into three categories: medical center, regional hospitals, and district hospitals and clinics. Hospital ownership was defined as public or private.

### Statistical analysis

Chi-squared and Student’s t tests were used to examine the differences between the late HCV group and control group. An unconditional logistic regression model was used to estimate the relative magnitudes of these differences. Odds ratio (OR) and 95% confidence intervals (CI) were calculated with the control group as the reference group. We identified trends in late HCV-related HCC diagnosis from 2012 to 2014 to 2017 to 2018. Next, we used multivariate logistic models to explore the predictors at both the individual and provider levels. All statistical analyses were performed using the SAS System for Windows Version 9.4 software (SAS Institute, Cary, NC, USA).

## Results

### Characteristics of the late HCV-related HCC group and control group

**Fig. 2:**
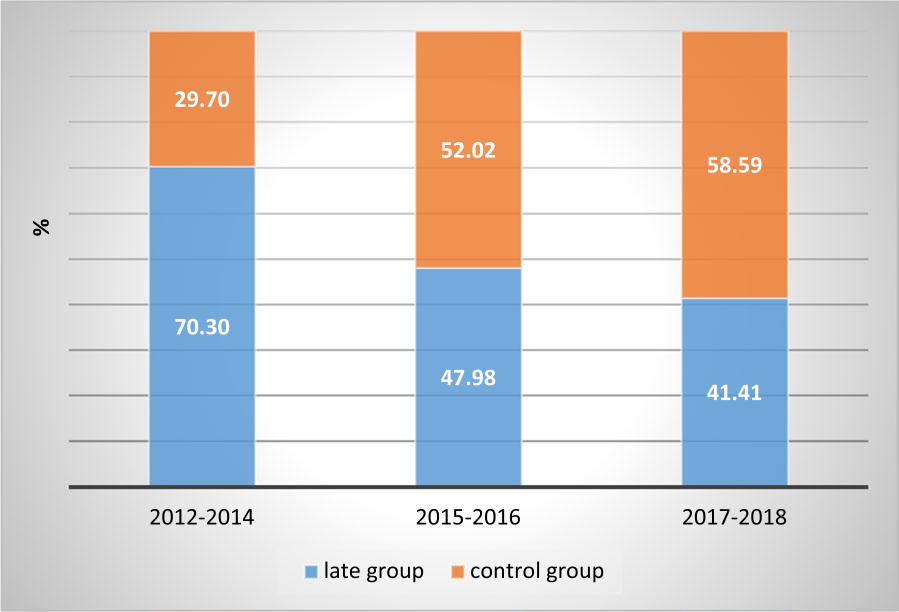
Trend in late diagnosis of hepatitis C virus related hepatocellular carcinoma between 2012–2014 and 2017–2018

Of all 6703 HCC patients who received the diagnosis during the study period, the proportion of late HCV-related HCC diagnoses decreased over time, from 70.30% between 2012 and 2014 to 41.41% between 2017 and 2018 (Fig. 2). Patients in the late group were significantly in male patients and patients older than those in the control group. In addition, compared with the control group, a higher proportion of patients in the late group had DM, alcohol-related disease, no routine medical checkups. A higher proportion of patients with late group were cared by, providers who were 55 years or older, female physician, and other specialties. Moreover, a lower proportion of patients with late group were union or association members, compared with the control group (Table 1).

**Table 1:** Demographic characteristics of patients with hepatitis C virus who were diagnosed late relative to hepatocellular carcinoma in Taiwan

Variables	Late group		Control group		*P* value
	n = 3733	%	n = 2970	%	
(A) Individual-level					
Gender					< 0.0001
Female	1483	39.73	1410	47.46	
Male	2250	60.27	1560	52.53	
Age	67.46	62.94	< .00001		
Co-morbidities					
DM	821	21.99	582	19.60	0.017
Dyslipidemia	16	0.43	18	0.60	0.310
Psychiatric disorders	19	0.51	16	0.54	0.867
Alcohol related disease	149	3.99	54	1.82	< 0.0001
Malignancy	95	2.54	55	1.85	0.057
SES					0.001
Low income group	817	21.89	538	18.11	
Union/association member	1871	50.12	1613	54.31	
< 60,000 (NTD$)	881	23.60	697	23.47	
≥ 60,000 (NTD$)	164	4.39	122	4.11	
Care seeking behavior					0.697
Very lower	944	25.29	730	24.58	
Lower	921	24.67	765	27.76	
Middle	944	25.29	731	24.61	
Higher	924	24.75	744	25.05	
Medical physical check-ups					< 0.0001
No	2707	72.52	1841	61.99	
Yes	1026	27.48	1129	38.01	
(B) Provider level					
Age			< 0.0001		
< 45	937	25.10	919	30.94	
45–54	1338	35.84	1236	41.62	
≥ 55	1458	39.06	815	27.44	
Gender					0.015
Female	252	6.75	158	5.32	
Male	3481	93.25	2812	94.68	
Specialty					< 0.0001
Internal medicine	1419	38.01	1258	42.36	
Family medicine	1039	27.83	851	28.65	
Other specialties	1275	34.15	861	28.99	
Accreditation status of practice					0.175
Clinics and district hospital	2326	62.31	1916	64.51	
Regional hospital	904	24.22	681	22.93	
Medical center	503	13.47	373	12.56	
Ownership of practice					0.682
Public	569	15.24	442	14.88	
Private	3164	84.76	2528	85.12	
(C) HCC diagnostic year					
2012	761	80.61	183	19.39	
2013	652	69.44	287	30.56	
2014	599	61.18	380	38.82	
2015	517	49.76	522	50.24	
2016	435	46.03	510	53.97	
2017	426	43.92	544	56.08	
2018	343	38.67	544	61.33	
2012–2014	2012	70.30	850	29.70	
2015–2016	952	47.98	1032	52.02	
2017–2018	769	41.41	1088	58.59	

DM = diabetes mellitus; Psychiatric disorders = including schizophrenia, depression, and anxiety; SES = socioeconomic status;

HCC = hepatocellular carcinoma; NTD = New Taiwan dollars; US$1 = approximately NT$30.24

Late group = late HCV diagnosis relative to hepatocellular carcinoma

### Factors and odds associated with late HCV-related HCC diagnosis

In the univariate analyses, the following factors were associated with significantly higher odds of late diagnosis: male patients (OR, 1.37; 95% CI, 1.24–1.51), patients aged 67 years or above (OR, 1.04; 95% CI, 1.04–1.05), with DM (OR, 1.16; 95% CI, 1.03–1.30), and alcohol-related disease (OR, 2.24; 95% CI, 1.64–3.07) (Table 2). By contrast, patients who had undergone a routine medical checkup were less likely to have a late HCV-related HCC diagnosis (OR, 0.62; 95% CI, 0.56–0.69). A dose response was observed between physician’s age and late HCV diagnosis. Patients who were treated by physicians aged 55 years or older had the highest odds of having a late HCV-related HCC diagnosis, followed by those cared by physicians aged 45–54 years (OR, 0.61; 95% CI, 0.54–0.68), and the last by physicians aged below 45 years (OR, 0.57; 95% CI, 0.50–0.65). Compared with patients who were mainly cared for by female physicians, patients who were mainly cared for by male physicians had a significantly lower risk of late HCV diagnosis (OR, 0.78; 95% CI, 0.63–0.95).

**Table 2:** Multiple logistic regression analysis of the predictive factors associated with late HCV among patients with a diagnosis of HCC

	Crude OR		Adjusted	
	OR	95%CI	OR	95%CI
Patient characteristics				
Gender (Male vs Female)	1.37	1.24–1.51	1.59	1.43–1.77
Age	1.04	1.04–1.05	1.06	1.05–1.06
Co-morbidities				
DM	1.16	1.03–1.30	1.18	1.04–1.34
Dyslipidemia	0.71	0.36–1.39	0.59	0.29–1.19
Psychiatric disorders	0.94	0.48–1.84	1.01	0.49–2.06
Alcohol related disease	2.24	1.64–3.07	2.95	2.11–4.13
Malignancy	1.38	0.99–1.94	1.41	0.99–2.01
SES				
Low income group	1.13	0.87–1.46	1.31	0.99–1.72
Union/association member	0.86	0.68–1.10	0.93	0.72–1.21
< 60,000 (NTD$)	0.94	0.73–1.21	1.15	0.88–1.51
≥ 60,000 (NTD$)	1.00		1.00	
Care seeking behavior				
Very lower	1.00		1.00	
Lower	0.93	0.81–1.07	0.85	0.73–0.98
Middle	1.00	0.87–1.14	0.82	0.70–0.95
Higher	0.96	0.84–1.10	0.73	0.62–0.85
Medical physical check-ups				
No	1.00		1.00	
Yes	0.62	0.56–0.69	0.58	0.52–0.65
**Provider characteristics**				
Age				
< 45	0.57		0.53	
45–54	0.61	0.50–0.65	0.62	0.47–0.61
≥ 55	1.00	0.54–0.68	1.00	0.55–0.70
Gender				
Female	1.00		1.00	
Male	0.78	0.63–0.95	0.70	0.56–0.87
Specialist				
Internal medicine	1.00		1.00	
Family medicine	1.08	0.96–1.22	1.06	0.93–1.21
Other specialties	1.31	1.17–1.47	1.28	1.13–1.45
Accreditation status of practice				
Clinics and district hospital	1.00		1.00	
Regional hospital	1.09	0.97–1.23	1.05	0.92–1.20
Medical center	1.11	0.96–1.29	1.02	0.86–1.20
Ownership of practice				
Public	1.00		1.00	
Private	0.97	0.85–1.11	1.03	0.89–1.19

DM = diabetes mellitus; Psychiatric disorders = including schizophrenia, depression, and anxiety; SES = socioeconomic status; HCC = hepatocellular carcinoma; NTD = New Taiwan dollars; US$1 = approximately NT$30.24

Compared with patients who received medical care primarily from internists, patients mainly cared for by other specialties had a significantly higher risk of having a late HCV diagnosis (OR, 1.31; 95% CI, 1.17–1.47) (Table 2).

After adjustment, the following factors were associated with significantly higher odds of late diagnosis: male patients (adjusted OR [AOR], 1.59; 95% CI, 1.43–1.77), patients aged 67 years or above (AOR, 1.06; 95% CI, 1.05–1.06), with DM (AOR, 1.18; 95% CI, 1.04–1.34), and alcohol-related disease (AOR, 2.95; 95% CI, 2.11–4.13) (Table 2). By contrast, patients who had more outpatient service utilization were significantly less likely to have a late HCV diagnosis. Compared to very lower group of outpatient service utilization, lower group (AOR, 0.85; 95% CI, 0.73–0.98), middle group (AOR, 0.82; 95% CI, 0.70–0.95), and higher group (AOR, 0.73; 95% CI, 0.62–0.85) were less likely to have a late HCV diagnosis. Patients who had undergone a routine medical checkup were less likely to have a late HCV diagnosis (AOR, 0.58; 95% CI, 0.52–0.65). Interestingly, significant variations were observed among providers. A dose response was observed between physician’s age and late HCV diagnosis. Compared to patients who were treated by physicians aged 55 years or older, less likely to have a late HCV diagnosis by those cared by physicians aged 45–54 years (AOR, 0.62; 95% CI, 0.55–0.70) and followed by those cared by physicians aged below 45 years (AOR, 0.53; 95% CI, 0.47–0.61). Compared with patients who were mainly cared for by female physicians, patients who were mainly cared for by male physicians had a significantly lower risk of late HCV diagnosis (AOR, 0.70; 95% CI, 0.56–0.87). Compared with patients who received medical care primarily from internists, patients mainly cared for by other specialties had a significantly higher risk of having a late HCV diagnosis (AOR, 1.28; 95% CI, 1.13–1.45) (Table 2).

## Discussion

Under the single-payer comprehensive universal health insurance program in Taiwan, the proportion of late HCV diagnoses has been decreasing over time. However, approximately 41% of patients still received a late HCV-related HCC diagnosis between 2017 and 2018. Continuing efforts are warranted to avoid missing the opportunity of earlier HCV diagnosis and developing serious liver disease.

In addition to factors repeatedly discussed in the literature (age, alcohol related disease, illicit drug use problems, health care–seeking behaviors, and SES), we identified additional patient and provider characteristics that were significantly associated with a higher risk of late HCV diagnosis. The first major risk factor was diabetes. Our findings illustrated that DM patients had a significantly higher risk of late HCV-related HCC diagnosis, regardless of sex and age. One would expect that DM patients tend to have more medical interactions and hence, their HCV are more likely to be detected earlier. One plausible explanation is that DM has the potential to accelerate processes associated with liver carcinogenesis, shortening the timeline in which HCV infection can lead to development of HCC [31, 32]. Regarding DM and the risk of HCV-related HCC, the relative risk (RR) of cohort studies have been shown to be 3.25 [33], and 3.1 [34] in Taiwan and 2.0 [35] and 1.73 [32] in Japan. This may be because DM is a crucial risk factor for nonalcoholic fatty liver disease [36] and nonalcoholic steatohepatitis [37], which increases the risk of HCC. Thus, DM patients require more active monitoring for HCC development [38], especially in the cirrhosis state [39]. Furthermore, DM may lead to more severe liver inflammation and fibrosis progression [40]. DM might be mediated by binding of insulin-like growth factor-1 (IGF-1) to the IGF-1 receptor and activate mitogen-activated protein kinase (MAPK) and phosphoinositide 3-kinase/protein kinase B (PI3K/AKT) signaling pathways leading to increased cell proliferation and decreased apoptosis [41]. Insulin resistance (IR), which has a negative impact on antiviral therapy responses [42] and liver-related outcomes [43], plays a crucial role in fibrosis progression. HCV infection promotes IR through the insulin signaling pathway in hepatocytes and lead to a rise in levels of tumor necrosis factor and interleukin-6 [44], which aid the advancement of liver steatosis and inflammation and subsequent cancer. Similar to the previous research [45], HCV-related fibrosis development was obviously accelerated in older patients and HCC were higher in older HCV patients, compared with younger patients. Thus, elderly DM patients should be considered the high-risk group for late HCV-related HCC.

HCC is more prevalent in men, which is associated with androgen receptor (AR) as a central role of sex preference [46, 47]. AR belongs to the superfamily of nuclear receptor [48, 48] and nuclear AR overexpression is allied with the progression of HCC [47]. To be specific, mammalian target of rapamycin (mTOR) signaling contributes a plausible molecular mechanism by virtue of enhanced AR protein stability through antagonizing proteasomal degradation and increasing nuclear localization [47]. Inhibition of AR induces AKT-mTOR signaling. Thus, targeting both AR and mTOR might be a promising therapeutic strategy for HCC, especially mTOR complex 1 (mTORC1), which facilitates AR nuclear localization and transactivation independently and together with androgen [46, 47]. HCV core protein also enhances AR-mediated signaling, which in turn upregulates vascular endothelial growth factor (VEGF) expression in hepatocytes and facilitates angiogenesis [49]. Immune system activation through the senescence-associated secretory phenotype (SASP), abnormal senescent cell accumulation, and pre-malignant hepatocytes evading senescence arrest lead to HCC occurrence [50]. HCV core protein bypasses normal stress-induced senescence by downregulating p16 [51]; furthermore, elevated T cell senescence and oxidative stress induced by HCV contribute to proliferation of pre-malignant cells [50]. Thus, a combination of pro-senescence therapy to stop tumor growth, with anti-senescence therapy to clear the senescent cells might be a convincing senescence-modulating treatment [52].

Another important risk factor is patients’ health care–seeking behaviors. Consistent with previous literature, we found that patients who had undergone a routine medical checkup or had more outpatient utilization were significantly less likely to have a late HCV diagnosis. In our study and the study by Samji et al. [22], a time period shorter than 2 or 3 years between the HCV diagnosis and occurrence of HCC was considered late diagnosis, both found that patients with a late HCV diagnosis had lower outpatient service utilization. Because HCC is likely to progress with no obvious initial symptoms, persons who were more attentive to their health or had more interactions with health care providers may have an increased likelihood of earlier diagnosis.

More importantly, our study found that provider characteristics play a crucial role in rates of late diagnosis. After adjusting for physician specialty and other variables, patients who were mainly cared for by female physicians had a significantly higher risk of late diagnosis. One plausible explanation may be the influence of patient-physician gender concordance on screening behaviors. For example, people who were care by male physician would yield a higher prostate cancer screening rate [53]. Female physicians were more likely to recommend human papillomavirus (HPV) vaccine [54] and mammography screening [55]. In our study, there were significantly more male than female HCV-related HCC patients (60.27% vs. 39.73%). Screening strategies shall take physician–patient gender concordance and more effective physician communication into consideration.

In addition, patients who were mainly cared for by older physicians or physicians with non-internal or family medicine specialties had a significantly higher risk of late diagnosis. Heterogeneity in education and training background of physicians, consensus of clinical practice guidelines and screening recommendation, awareness of HCV infection across different age cohorts, and specialty of physicians may have contributed to the variation in their practices.

Our study has several limitations. First, unlike performing liver biopsies to indicate cirrhosis or using mean fibrosis 4 (FIB 4) score for late HCV diagnosis [19], using a 3-year interval as the cutoff for late diagnosis may seem arbitrary. The previous research showed that the DAA group had a significantly higher survival rate than the non-DAA group during 3 years follow up [56]. Another research disclosed the potentially lower risk of severe liver disease among patients with SVR after anti-viral therapy duration of 3-years post-treatment time [57]. In addition, more than half of patients developing HCC within 3 years between HCV diagnosis and HCC diagnosis in our data; therefore, a 3-year interval was used as the cut-off time period for defining ‘‘late” diagnosis. Moreover, because in a typical disease course it takes approximately 20 to 30 years or longer for HCV infection to develop into HCC, our result may be biased toward the null. The true magnitude of late diagnosis may be much higher than that observed. We also performed the sensitivity analysis using 2-year and 5-year time lag (data not shown) as the cut-offs and the main results remained robust. Second, the use of ICD-9/10-CM codes may yield potential misclassification bias. Therefore, to reduce potential misclassification bias, we used the HCC recorded in the registry of the Catastrophic Illness Patient Database as our end point, not liver cirrhosis. This is because the NHI program requires a histologic confirmation and undertakes a review process for officially recording a diagnosis of liver cancer in in the registry of the Catastrophic Illness Patient Database. Third, patients with drug abuse and HIV have not been analyzed due to a small sample size. Fourth, due to the inherited limitations of claims data, some important confounding factors such as education and clinical symptoms may not be perfectly controlled in the analyses. Residual confounding bias may be possible.

This population-based study with a large number of HCC cases allows us to provide robust estimates of risk factors associating with delayed diagnosis of HCV infection. However, continued efforts in narrowing the gaps for effective secondary prevention and treatment are needed [58]. Taiwan has implemented a policy to improve treatment access to reduce DAA cost to approximately US$750 for a 12-week therapy course. Taiwan HCV screening policy is that people more than 45 years-old and aboriginals more than 40 years-old had the once in a lifetime to receive HCV screening. Early diagnosis and identification of patients is critical for effective decision-making for HCV treatment. Robust governmental action, such as in the case of Taiwan, is warranted to eliminate HCV before 2030 [58]. Similar to the policies such as time testing for individuals born between 1945 and 65 [59, 60] and risk-based screening for illegal drug users [61], routine HCV screening in high-risk groups such as DM and alcohol related disease may help reduce the late HCV diagnosis. Timely diagnosis and treatment for HCV related HCC was suboptimal at the population level. The shorter the length of time between HCV and HCC diagnoses might represent poor HCV management. If DM patients were to diagnose HCV early enough, more timely diagnosis of cirrhosis and early screening and treatment intervention care then proceed, and reduce the risk of the patient developing HCC in the first place. Hospital-based HCV screening [62, 63] for elderly DM patients should be considered as a model for early diagnosis.

## Conclusions

We recommend that elderly and patients who have DM and alcohol related disease receive early HCV screening and reduce the incidence of inoperable HCC. The finding also highlights the role of health care providers in improving early detection of HCV [27].

## Supplementary Information

**Additional file 1**. Appendix A. Diagnosis codes. Table A Codes of International Classification of Diseases, 9/10th Revision, Clinical Modification (ICD-9/10-CM).

## Data Availability

Data are available from the Collaboration Center of Health Information Application of the Ministry of Health and Welfare. The data utilized in this study cannot be made available in the paper, the supplemental files, or in a public repository due to the ‘‘Personal Information Protection Act’’ executed by Taiwan’s government, starting from 2012. Requests for data can be sent directly to the Collaboration Center of Health Information Application of the Ministry of Health and Welfare (https://dep.mohw.gov.tw/dos/cp-5283-63826-113.html).

## Abbreviations

HCV: Hepatitis C virus
DAA: Direct-acting antiviral
HCC: Hepatocellular carcinoma
ESLD: End stage liver disease
SES: Socioeconomic status
HIV: Human immunodeficiency virus
NHIRD: National Health Insurance Research Database
ICD-9/10- CM: International Classification of Diseases, 9th/10th Revision, Clinical Modification
HBV: Hepatitis B virus
ESRD: End stage renal disease
DM: Diabetes mellitus
NT: New Taiwan dollars
OR: Odds ratio
95% CI: 95% Confidence intervals
AOR: Adjusted odds ratio
RR: Relative risk
IGF-1: Insulin-like growth factor-1
MAPK: Mitogen-activated protein kinase
PI3K: Phosphoinositide 3-kinase
AKT: Protein kinase B
IR: Insulin resistance
AR: Androgen receptor
mTOR: Mammalian target of rapamycin
mTORC1: MTOR complex 1
VEGF: Vascular endothelial growth factor
SASP: Senescence-associated secretory phenotype
HPV: Human papillomavirus
FIB 4: Fibrosis 4
