# Supplementary material for: Late hepatitis C virus diagnosis among patients with newly diagnosed hepatocellular carcinoma: a case–control study
Source: BMC Gastroenterol. 2022 Sep 17;22:425. doi: 10.1186/s12876-022-02504-6 (PMC9482748; doi:10.1186/s12876-022-02504-6)
Supplement: Supplementary file 1 — Additional file 1. Appendix A. Diagnosis codes. Table A Codes of International Classification of Diseases, 9/10th Revision, Clinical Modification (ICD-9/10-CM). [file 12876_2022_2504_MOESM1_ESM.pdf]

Appendix A. Diagnosis codes

Table A Codes of International Classification of Diseases, 9/10th Revision, Clinical Modification (ICD-9/10-CM)

Diseases	ICD 9/10	Code
HCC	ICD 9	155.0,155.2
	ICD 10	C22.0, C22.2, C22.3, C22.4, C22.7, C22.8, C22.9, Z51.12
HCV	ICD 9	"07041","07044","07051","07054","V0262"
	ICD 10	B17.11, B19.21, B18.2, B17.10, B19.20, B18.2, Z22.52
HBV	ICD 9	"0702","0703","V0261","V0269","V0261" "07022","07032","V0269
	ICD 10	B16.2, B19.11, B16.0, B18.1, B18.0, B16.9, B19.10, B16.1, B18.1, B18.0, Z22.51, Z22.59, Z22.51, B18.1, B18.1, Z22.59
Diabetes	ICD 9	250
	ICD 10	E08, E09, E10, E11, E13
ESRD	ICD 9	"585","586"
	ICD 10	N18.4, N18.5, N18.6, N18.9, N19
Other hepatitis	ICD 9	"5731", "5732", "5733
	ICD 10	K77, K77, K71, K75
dyslipidemia	ICD 9	272.0, 272.1, 272.2, 272.3, 272.4
	ICD 10	E78.0, E78.1, E78.2, E78.3, E78.4, E78.5
Alcoholic related disease	ICD 9	291, 303, 305.0, 571.0, 571.1, 571.2, 571.3, 303.0, 303.9
	ICD 10	F10, K70.0, K70.1, K70.2, K70.3, K70.4, K70.9
Malignancy	ICD 9	140-199, 209, 230-239, 200-208, but exclude 155.0,155.2
	ICD 10	C00-D49, but exclude D10-D36, D3A, C22.0, C22.2, C22.3, C22.4, C22.7, C22.8, C22.9, Z51.12
Schizophrenia	ICD 9	295.0–295.9, 298.8–298.9, 297.1–297.3
	ICD 10	F20–F29
Depression	ICD 9	296.0–296.1, 296.4–296.8, 296.2, 296.3, 300.4, 311, 296.9
	ICD 10	F30, F31, F34.0, F32, F33, F34.1, F38.1,

		F34.8, F34.9, 38.0, F38.8, F39
Anxiety disorders	ICD 9	300.0, 300.2, 300.3, 309.8; 308.3, 309.0, 309.1
	ICD 10	F40, F41, F42, F93.0–F93.2; F43.0, 43.1, 43.8, 43.9
